# GRE: A Framework for Significant SNP Identification Associated with Wheat Yield Leveraging GWAS–Random Forest Joint Feature Selection and Explainable Machine Learning Genomic Selection Algorithm

**DOI:** 10.3390/genes16101125

**Published:** 2025-09-24

**Authors:** Mei Song, Shanghui Zhang, Shijie Qiu, Ran Qin, Chunhua Zhao, Yongzhen Wu, Han Sun, Guangchen Liu, Fa Cui

**Affiliations:** 1 School of Mathematics and Statistics, Ludong University, Yantai 264025, China; ytsongmei@163.com (M.S.);; 2 School of Horticulture, Ludong University, Yantai 264025, Chinayongzhenwu1204@163.com (Y.W.);; 3 Yantai Key Laboratory of Molecular Breeding for High-Yield and Stress-Resistant Crops and Efficient Cultivation, Yantai 264000, China

**Keywords:** genomic selection, explainable machine learning, wheat yield, GWAS, random forest, SHAP

## Abstract

**Background**: Facing global wheat production pressures such as environmental degradation and reduced cultivated land, breeding innovation is urgent to boost yields. Genomic selection (GS) is a useful wheat breeding technology to make the breeding process more efficient, increasing the genetic gain per unit time and cost. Precise genomic estimated breeding value (GEBV) via genome-wide markers is usually hampered by high-dimensional genomic data. **Methods**: To address this, we propose GRE, a framework combining genome-wide association study (GWAS)’s biological significance and random forest (RF)’s prediction efficiency for an explainable machine learning GS model. First, GRE identifies significant SNPs affecting wheat yield traits by comparison of the constructed 24 SNP subsets (intersection/union) selected by leveraging GWAS and RF, to analyze the marker scale’s impact. Furthermore, GRE compares six GS algorithms (GBLUP and five machine learning models), evaluating performance via prediction accuracy (Pearson correlation coefficient, PCC) and error. Additionally, GRE leverages Shapley additive explanations (SHAP) explainable techniques to overcome traditional GS models’ “black box” limitation, enabling cross-scale quantitative analysis and revealing how significant SNPs affect yield traits. **Results**: Results show that XGBoost and ElasticNet perform best in the union (383 SNPs) of GWAS and RF’s TOP 200 SNPs, with high accuracy (PCC > 0.864) and stability (standard deviation, SD < 0.005), and the significant SNPs identified by XGBoost are precisely explained by their main and interaction effects on wheat yield by SHAP. **Conclusions**: This study provides tool support for intelligent breeding chip design, important trait gene mining, and GS technology field transformation, aiding global agricultural sustainable productivity.

## 1. Introduction

The birth of the genomic selection (GS) technology marks a paradigm shift in crop breeding from “experience-driven” to “data-driven”. Since Meuwissen et al. [1] first proposed the concept of GS, this technology has significantly shortened the breeding cycle and improved genetic gain by using genome-wide markers to predict the genomic estimated breeding value (GEBV) of individuals [2,3]. In wheat breeding, the application of GS can be traced back to the pioneering research of Crossa et al. [4]. They verified the feasibility of GS in predicting wheat yield traits through simulation data. Subsequently, the field experiments of Dreisigacker et al. [5] further confirmed that the annual genetic gain of spring wheat yield can be significantly improved by using rapid recurrent genomic selection, which far exceeds the accuracy of traditional phenotypic selection. The core of this breakthrough is that GS does not require precise QTL mapping, and prediction can be achieved only through the linkage disequilibrium between markers and traits, especially for complex traits such as yield that are controlled by multiple genes [6].

In recent years, with the popularization of high-throughput sequencing technology, the density of wheat whole-genome SNP markers has increased from 9 K in the early stage to 660 K [7], providing a richer genotype data foundation for GS. However, the surge in the number of markers has also brought about the “curse of dimensionality”, i.e., when the number of markers far exceeds the sample size, the model is prone to overfitting, and the computational cost increases exponentially [8], which greatly limits the large-scale application of GS in breeding practice. Therefore, developing efficient feature selection methods to reduce the marker set has become a key breakthrough for improving the efficiency of GS.

As one of the most important food crops in the world, the genetic dissection of yield traits in wheat has always been the core of molecular breeding research. Yield is jointly determined by its four component traits, namely, grain yield (GY), thousand-grain weight (TKW), effective tiller number (ETN), and grains per spike (GPS). Each trait is controlled by multiple genes and exhibits complex epistatic/interaction effects [9]. For example, the heritability of TKW is approximately 0.6–0.7, but about 30% of its phenotypic variation is contributed by minor quantitative trait loci (QTLs) [10]. This complexity makes traditional marker-assisted selection (MAS) difficult to implement effectively. Early studies have shown that when only a few major QTLs are used for MAS, the prediction accuracy of wheat yield is usually low, while GS can effectively improve the accuracy by integrating genome-wide markers [11,12]. However, even so, high-dimensional SNP data still pose two major challenges: one is that redundant markers lead to “noise amplification” in the model. For example, the random fluctuations of non-associated SNPs may mask the true association signals; the other is that the consumption of computational resources is too large, and existing breeding platforms can hardly afford the routine analysis of millions of markers [12]. Therefore, how to retain significant markers and eliminate redundant information through feature selection has become the core issue for improving the accuracy and efficiency of GS.

Current feature selection methods can be divided into two categories: methods based on statistical association (such as GWAS) and methods based on machine learning (such as random forest). GWAS screens SNPs by testing the significant association between markers and traits [12]. There is evidence that using GWAS-derived SNP markers in genomic prediction (GP) offers advantages [13,14,15,16]. Shi et al. found that significant GWAS-derived SNPs could raise predictive ability (PA), attaining a high r value of 0.84, which was far greater than the PA from randomly chosen SNP sets [14]. Zhang et al. demonstrated that combining GWAS-related markers with machine learning can enhance the accuracy of predicting fall dormancy in alfalfa [13]. Jiang et al. found that GWAS-derived markers combined with machine learning algorithms improved the efficiency of GP in alfalfa yield. When using all markers for prediction, the prediction accuracy is between 0.11 and 0.18, while after combining GWAS-derived markers, the prediction accuracy can be increased to between 0.7 and 0.8. This finding provides strong support for the efficient selection of alfalfa root traits [15].

Machine learning methods identify markers with significant predictive contributions through a data-driven approach. Shahsavari et al. employed several feature selection methods, including principal component analysis (PCA), stepwise selection (SS) [17], Pearson correlation coefficient, and least absolute shrinkage and selection operator (LASSO) regression [18], to reduce the number of yield-related traits and identify the most effective traits that can explain the variation in seed yield (SY) [19]. Heinrich et al. investigated an incremental feature selection method for ranking single-nucleotide polymorphisms (SNPs) based on genome-wide association study results [20]. They combined this method with random forest as a prediction model and applied it to multiple animal and plant datasets [21]. However, machine learning models may overfit to noisy markers, leading to insufficient biological interpretability of the results [22].

Most existing studies adopt a single feature selection method, and the models are relatively limited to linear models (such as GBLUP). Chachar et al. reviewed the strategy of genomic selection and its impact on breeding programs for improving wheat yield [23]. Alam et al. [24] emphasized the current opportunities in wheat breeding and for the development of future wheat cultivars. Although Wang et al. [25] verified the advantages of deep learning models, they did not systematically analyze the impact of the scale of feature subsets. Finally, the interpretability of the GS model is very important for helping breeders screen significant SNPs for wheat yield. Currently, there is little research in this area [6,26], and in-depth and systematic research is urgently needed.

In response to the above limitations, this study proposes GRE, a “GWAS + random forest” hybrid feature selection with an explainable GS framework. By integrating the advantages of the two methods, a marker subset with both biological significance and predictive ability is constructed. The core content of the study includes the following: (1) in terms of multi-dimensional feature subset design, systematically compare the intersection, union, and individual subsets of markers screened by GWAS and RF, and analyze the impact of marker sets of different scales on prediction; (2) in terms of multi-model collaborative verification, incorporate one classical GS model and five machine learning algorithms simultaneously to comprehensively evaluate the gain effect of feature selection on different types of models; (3) in terms of comprehensive performance evaluation, accuracy metrics (PCC, *R^2^*) and error metrics (MSE, MAE) are leveraged to evaluate GS models’ performance; and (4) in terms of model interpretability, leverage SHAP techniques combined with XGBoost technology to systematically quantify and visually display information such as the main and interaction effects of SNPs, and the impact of samples (breeding lines) on the breeding accuracy of GS at both macro and micro levels, assisting in understanding the core role of significant SNPs in wheat yield traits.

## 2. Materials and Methods

**Figure 1:**
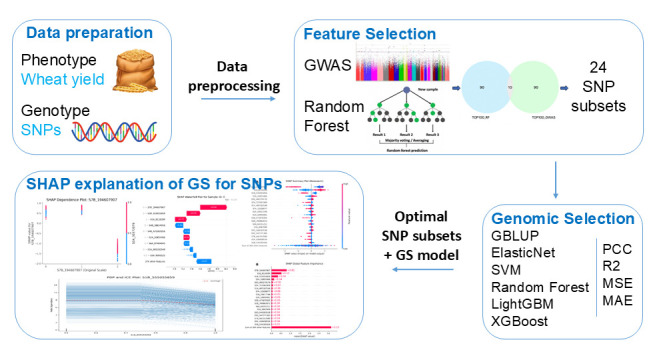
Illustration of the GRE framework for significant SNP identification in wheat yield.

The main procedures of GRE consist of data preparation, feature selection, genomic selection, and SHAP explanation for the GS model (Figure 1). The main objectives of the GRE framework include the following: (1) verifying the enhancing effect of the combined feature selection of “GWAS + RF” on the prediction accuracy of GEBV for wheat yield; (2) determining the optimal scale of the feature subset (such as TOP 500) and the best combination of prediction models; and (3) clarifying the interpretability of the GS model based on feature selection. By achieving these objectives, the research will provide efficient marker screening and model selection schemes for wheat molecular breeding and promote the transformation and application of GS technology from the laboratory to the breeding field. The details of GRE are as follows (Figure 1).

### 2.1. Data Sources and Preprocessing

In this study, a winter wheat population consisting of 1,768 breeding lines [27] was utilized. Ten winter wheat breeding populations adapted to the US Pacific Northwest were employed, including AMP, two F5 populations, two doubled haploid (DH) biparental populations, and five Prel validation populations, with their planting times and locations specified [27]. SNP markers were screened to obtain a minor allele frequency (MAF) > 0.05 and 10% missing data. After quality control, 11,089 SNPs were obtained, and their physical positions were designated. The linkage disequilibrium k-nearest neighbor imputation (LD kNNi) function in TASSEL v.5.2.25 was used to impute the missing data. For GS calculations, the genotype data were converted into numerical form codes as 0, 1, and 2.

### 2.2. Feature Selection Strategy of GRE

GWAS feature selection strategy: After filtering and quality control, 11,089 markers were retained and used for GWAS. The fixed and random model circuitous probability unification (FarmCPU) model was employed to control the population structure. The GAPIT3 v3.0 software (https://zzlab.net/GAPIT/, 22 September 2025) was used to calculate the association *P*-values between SNPs and yield [28]. The SNPs were sorted in ascending order of the *P*-values, and the TOP 100/200/500/1000/2000/3000 SNPs were selected.

Random forest feature importance: The RandomForestRegressor in the Python scikit-learn v1.4.2 package (https://scikit-learn.org/stable/modules/generated/sklearn.ensemble.RandomForestRegressor.html#sklearn.ensemble.RandomForestRegressor, 22 September 2025) was used to build a regression model, calculate the Gini coefficient as the feature importance score [29], sort the scores from high to low, and select the TOP 100/200/500/1000/2000/3000 SNP.

Subset construction: Six intersection subsets and six union subsets were constructed using the TOP 100/200/500/1000/2000/3000 SNP subsets of GWAS and RF. In addition, there were 12 individual SNP subsets, namely the TOP subsets of GWAS and RF, respectively, and one original complete set (with 11,089 SNPs), totaling 25 subsets (Supplementary Table S2).

### 2.3. Prediction Model and Hyperparameter Settings

GBLUP model: The BWGS v0.2.1 package (https://cran.r-project.org/web/packages/BWGS/index.html, 22 September 2025) was utilized to implement the genomic best linear unbiased prediction (GBLUP) [30].

Machine learning models: ElasticNet, SVM, random forest, LightGBM, and XGBoost regression calculations were implemented using the Python scikit-learn v1.4.2 package (https://scikit-learn.org/stable/supervised_learning.html, 22 Septermber 2025). The hyperparameter tuning list is presented in Supplementary Table S1.

### 2.4. Model Training and Validation

Data partitioning: The original dataset (breeding lines) was randomly divided into a training set and a test set in an 8:2 ratio. This step was repeated five times to reduce sampling error.

Cross-Validation: Five-fold cross-validation and Bayesian optimization were used on the training set to find the best parameters and optimize the hyperparameters (Supplementary Table S1).

Model evaluation metrics

In this study, we introduce two groups of model evaluation metrics, including prediction accuracy (PCC, *R^2^*) and error (MSE, MAE). The definitions of the metrics are presented in Supplementary Method S1.

### 2.5. Comparison of Random SNP Selection with Feature Selection Using GRE

To verify the GRE feature selection performance proposed in this paper, as a control, the same number of feature SNPs was randomly selected, for a total of 17 scenarios (Supplementary Table S2). The GRE features—selected using the intersection, single subset (for example, the TOP 100 SNPs selected only by GWAS), and the union of RF and GWAS—were tested with various methods to verify the advantages of the proposed GRE feature selection. Random selection was performed three times to overcome contingency, and the calculation parameters for each iteration were set as in Section 2.4.

### 2.6. GRE’s Explainability Leveraging SHAP Techniques

To understand the explanatory power of significant SNPs for wheat traits, taking XGBoost as an example, combined with the SHAP model interpretation technique [26,31], we explore “black box” models such as complex ensemble learning, and analyze the biological associations between SNPs and traits. The SHAP analysis process starts with calculating the core SHAP values, and then provides a global and macroscopic feature insight through a summary plot, that is, analyzing the contribution of all SNPs to wheat yield traits; then, it reveals the complex interactions among significant SNP features through a dependence plot; it further uses a waterfall plot to precisely decompose the prediction of a single sample at the micro level; and finally, it combines the partial dependence plot (PDP) and the individual conditional expectation (ICE) plots to verify the average and individual effects of SNP features. Among them, the PDP shows the impact of SNP features on the average GEBV prediction results of the model, and the ICE plot evaluates the impact of SNP features on the prediction results of each breeding line.

### 2.7. Computing Platform

This paper uses the following platform configuration: Intel(R) Core (TM) i7-14700KF, 3.40 GHz, RAM 64.0 GB, Python 3.12, R-4.2.3, scikit-learn 1.4.2.

## 3. Results

### 3.1. The Dimension of the Feature Subset and the Label Distribution

**Figure 2:**
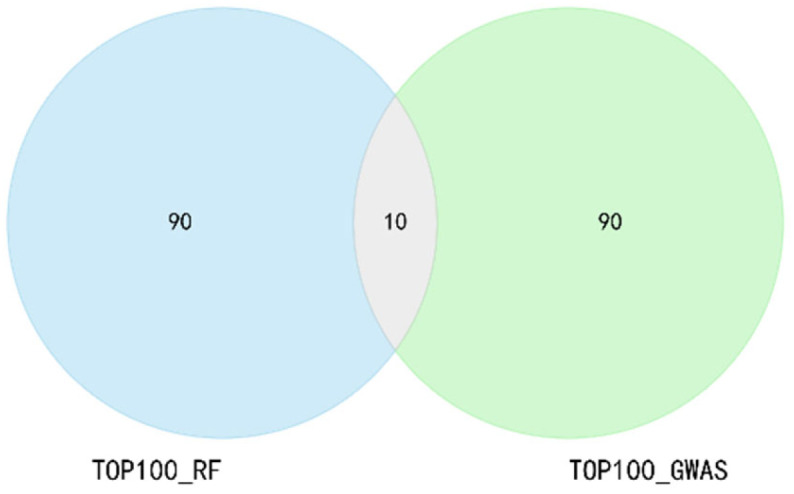
Intersection and union of the TOP 100 subsets of RF and GWAS. The intersection subset contains 10 SNPs, and the union contains 190 SNPs.

The intersection rate of the TOP subsets of GWAS and RF (10/100, 17/200, 43/500, 120/1000, 383/2000, 821/3000) is approximately 9–27%, and the number of markers in the union subset (190/100, 393/200, 957/500, 1880/1000, 3617/2000, 5179/3000) is 1.73–1.97 times that of the corresponding TOP subset, thereby retaining more potentially associated markers. Taking the TOP 100 of GWAS and RF as an example (Figure 2), the intersection subset contains 10 SNPs, and the union is 190 SNPs. The number of SNPs in all intersection and union subsets is shown in the Supplementary Table S2.

### 3.2. The Impact of Feature Selection Strategy on Prediction Accuracy

(1).  Comparison of the effects of random SNP selection and GRE in predicting GEBV

**Figure 3:**
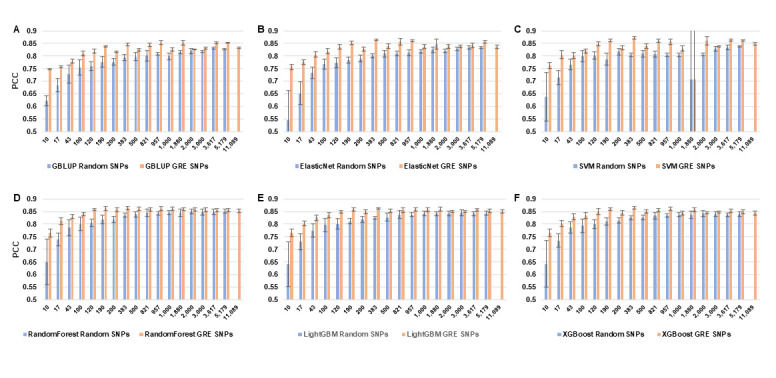
The comparisons of PCC between randomly selected SNPs and GRE SNPs for six GS models on subsets of different SNP dimensions. The height of the bar chart represents the mean of the PCC of repeated tests, and the error bars represent the standard deviation. The last bar in each sub-plot is the calculation result of all 11,089 SNPs, which serves as a reference. (**A**) GBLUP model. (**B**) ElasticNet model. (**C**) SVM model. (**D**) Random forest model. (**E**) LightGBM model. (**F**) XGBoost model.

We used six methods, including GBLUP and five machine learning methods, to test and compare the prediction accuracy (PCC) of GEBV under two feature selection strategies: (1) using GRE, that is, features selected by the joint strategy of the intersection, individual, and union of RF and GWAS; (2) random selection of the same number of features as (1). The results show that in all SNP subset scenarios with the identical SNP number, the accuracy (PCC) of any model constructed by randomly selected SNPs is lower than that of the model corresponding to the GRE feature selection strategy proposed in this study (Figure 3, Supplementary Table S2), and the error (standard deviation) is also smaller for GRE, which means the GRE-selected SNPs help GS models obtain more robust predictions. Furthermore, we find that in five out of six of the models’ results, the accuracy is low with a small number of SNPs, and it stabilizes as the number of SNPs increases to a certain level, and then decreases. The features of all 11,089 SNPs are not necessarily conducive to the highest accuracy, indicating redundant SNPs among all SNPs. When the number of SNPs is lower than around 383, the prediction accuracy of GEBV is increased by about 5–23% on average. When the number of SNPs is greater than 383, the increase is about 1–4% (Supplementary Figure S1).

(2).  Comparison of PCC prediction results between the dimensionality-reduced subset and the whole SNP set

**Figure 4:**
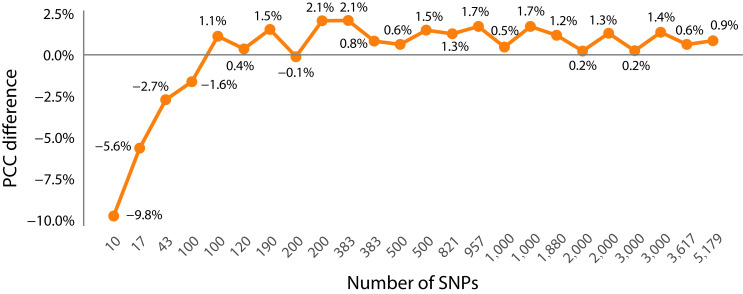
The difference between the average predicted PCC of the GS model under different SNP dimensions and the prediction using all 11,089 SNPs.

**Figure 5:**
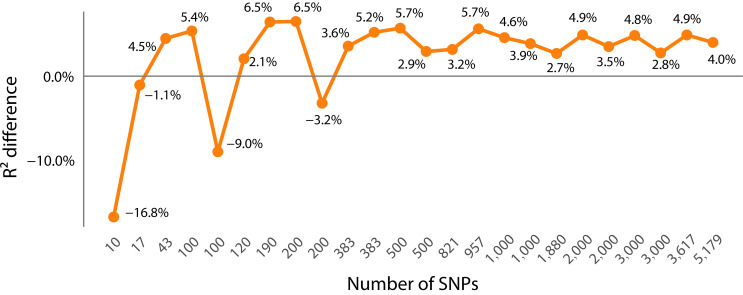
The difference between the average predicted *R*^2^ of the GS model under different SNP dimensions and the prediction using all 11,089 SNPs (GBLUP and SVM are unstable and not included in the calculation).

**Figure 6:**
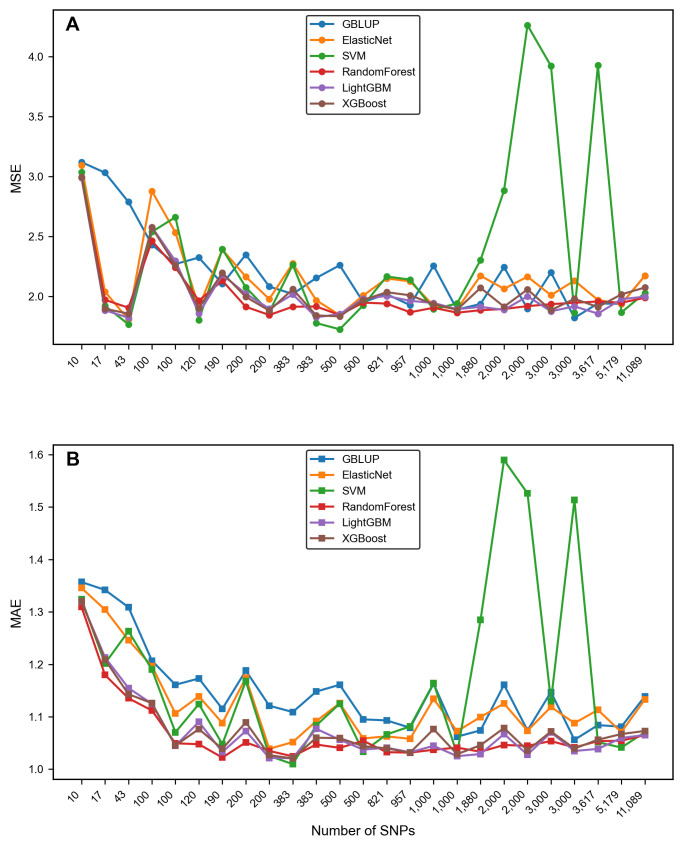
MSEs and MAEs of GS models on different SNP subsets. (**A**) MSEs of six GS models. (**B**) MAEs of six GS models.

**Figure 7:**
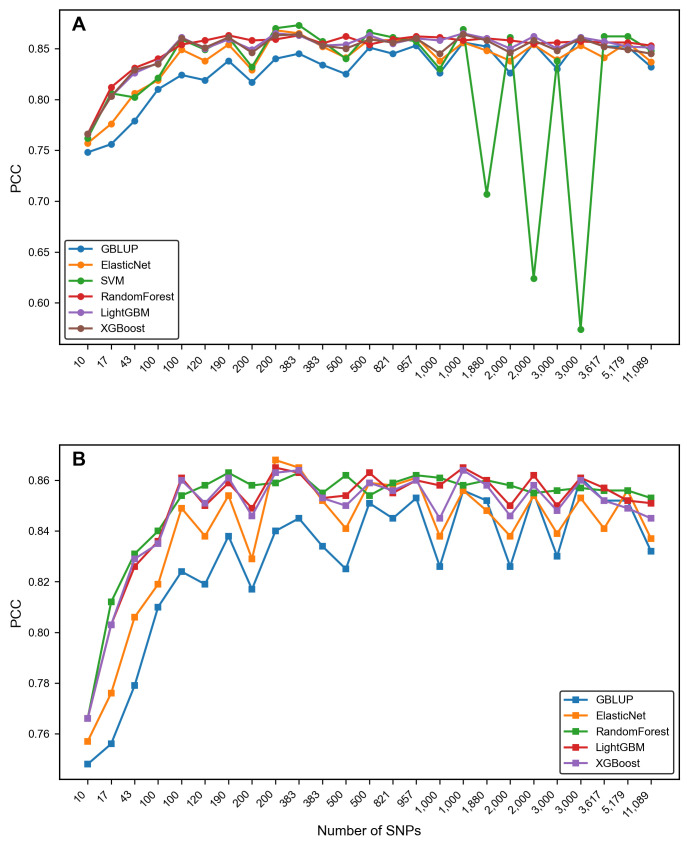
Comparison of the PCC of different GS models on all subsets and the whole set. (**A**) The SVM algorithm is included; (**B**) the SVM algorithm is excluded.

The PCC of most dimensionality-reduced subsets (with the number of SNPs > 100) is higher than that of the whole SNP set (with an average increase of approximately 0.2–2.1%) (Figure 4). That is, when the number of SNPs is lower than about 100, the PCC of the subset prediction is lower than that of the entire set of SNPs. However, when the number of SNPs in the subset increases, the average PCC of each algorithm (except the SVM algorithm) for SNPs of other dimensions is higher than that of using the entire set of SNPs (Figure 4). The goodness-of-fit index *R*^2^ of the model also shows the same situation as PCC (Figure 5) (the *R*^2^ of GBLUP and SVM is unstable and not included in the calculation). The errors decrease along with the increase in SNP dimensions (Figure 6, see details in Section Comparison of the error metrics). Among them, the union of “GWAS + RF” TOP 200 subset (383 SNPs) performs the best (average PCC = 0.86), which is higher than the individual subsets of single methods, i.e., GWAS TOP 200, RF TOP 200 (0.85, 0.84), and is 2.1% higher than the PCC of using the whole set. From the perspective of a single subset, GWAS is superior to RF (Supplementary Table S2, Figure 7B), indicating that the GWAS model can better incorporate the relationships between genotypes and phenotypes. In addition, the SVM algorithm performs relatively stably and has a high prediction accuracy in small subsets (≤1000 SNPs) (average PCC = 0.842). However, it performs unstably in large subsets (>1000 SNPs) (average PCC = 0.772) (Figure 6 and Figure 7A), which is lower than the PCC obtained using all 11,089 SNPs (PCC = 0.82). In summary, the results indicate that there is information redundancy in the whole 11,089 SNP set, and the GRE feature selection strategy proposed in this paper is of great significance for improving the accuracy of subsequent models.

(3).  Comparison of the error metrics

The error metrics (MSE, MAE) are negatively correlated with dimension (except for SVM in the large subset of 2000+ SNPs) (Figure 6). That is, as the dimension of SNPs increases, both MSE and MAE decrease and tend to stabilize. On subsets with a dimension greater than 200 SNPs, the MSEs of the RF, LightGBM, and XGBoost algorithms are reduced by approximately 7–15% compared to those of the entire SNP set.

### 3.3. Comparison of Model Performance

In terms of prediction accuracy, random forest and LightGBM performed best on most subsets (average PCC = 0.849–0.851) (Figure 7B, Supplementary Table S3), followed by XGBoost (average PCC = 0.847) and ElasticNet (average PCC = 0.84). These four machine learning models were significantly higher than GBLUP (average PCC = 0.829). Among the machine learning models, SVM generally performed better on small subsets (<1000 SNPs), but was the most unstable on large subsets (>1000 SNPs) (Figure 7A).

### 3.4. Identification of the Optimal Combination

The best prediction combination is the union of GWAS TOP 200 and RF TOP 200 subset (383 SNPs) + XGBoost/ElasticNet, which performs optimally in terms of comprehensive PCC (>0.864) (Supplementary Table S3), MAE (<1.05) (Figure 6B). In terms of stability, the standard deviation (SD) of the PCC of this combination across five repetitions is lower than 0.005, which is lower than that of other combinations (Supplementary Table S3).

### 3.5. Model Interpretability Based on SHAP

Taking XGBoost in the union of RF TOP 200 and GWAS TOP 200 (including 383 SNPs) as an example, we leveraged the SHAP interpretation techniques to analyze the XGBoost “black box” model and explore the biological associations between significant SNPs and wheat yield traits.

**Figure 8:**
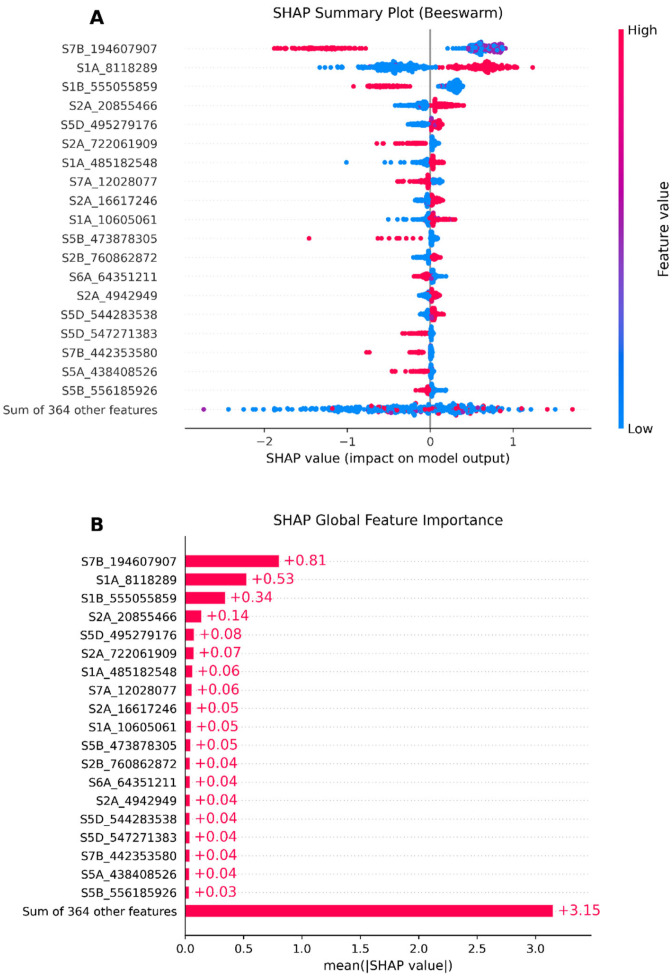
SHAP summary plot series (global feature analysis). (**A**) The Beeswarm plot shows the impact of the magnitude of feature values on the SHAP values. The X-axis represents the SHAP value, and the importance of SNPs along the Y-axis decreases from top to bottom. Each point in the figure represents a breeding line (sample), and the color indicates the magnitude of the feature value of the sample point. Red represents allele = 2, purple represents allele = 1, and blue represents allele = 0. (**B**) SHAP global feature importance shows the importance of each characteristic SNP on wheat yield traits from a global perspective.

First, the SHAP baseline value (expected value) was calculated to be 7.858. Based on this result, a summary plot was drawn to provide a global and macroscopic feature insight, that is, to analyze the contribution of the 383 SNPs to the wheat yield traits. Taking the TOP 20 among the 383 SNPs as an example, we examine the Beeswarm plot (Figure 8A) to see how the SNP S7B_194607907 allele values affect the SHAP value. For example, when SNP S7B_194607907’s allele is 0 or 1, this SNP might have a promoting effect on wheat yield, and when its allele is 2, it might inhibit yield improvement, and so on. Figure 8B shows the global feature importance. The average absolute value of the SHAP value of each feature is calculated and then sorted by size. The longer the bar, the greater the average impact of this feature on the model prediction. Similarly, SNP S7B_194607907 has the greatest feature importance, and so on. In brief, when the SNP S7B_194607907’s allele is 0 or 1, it has the main effect of promoting wheat yield, and the same applies to the other SNPs.

**Figure 9:**
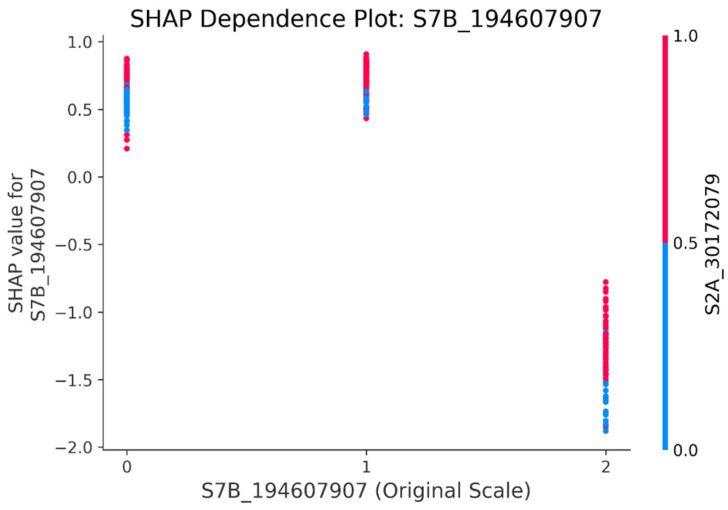
Dependency plot of SNP S7B_194607907 and S2A_30172079 (SNP interaction analysis). The X-axis represents the original value of the main feature (here, the allelic values of the SNP, 0, 1, and 2), and the Y-axis is the SHAP value of the main SNP, i.e., SNP S7B_194607907.

Second, the complex interactions among significant SNP features are revealed through the dependence plot. The dependence plot (Figure 9) shows how the value of a single SNP (the main effect), for example, SNP S7B_194607907, affects its own SHAP value, and can also reveal its interaction effect with other features (such as SNP S2A_30172079 in Figure 9) through color. There is a clear vertical color separation in the figure (for example, the upper part is mainly red dots, and the lower part is mainly blue dots), indicating that the effect of the main SNP S7B_194607907 may be strongly influenced by the interacting feature SNP S2A_30172079.

**Figure 10:**
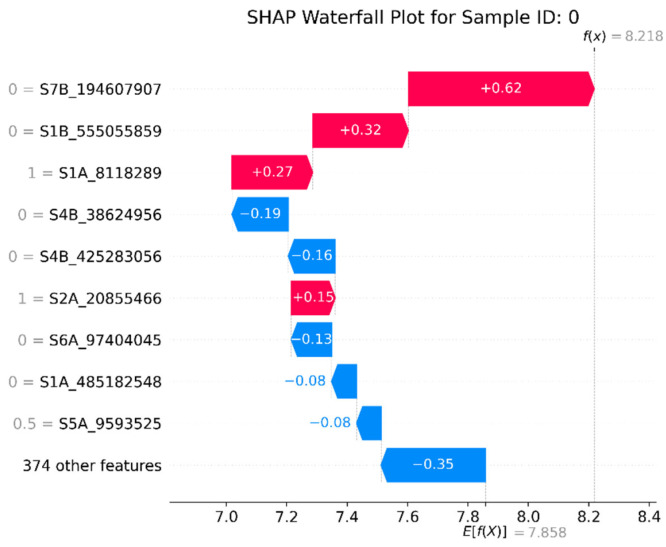
SHAP waterfall plot (single sample No. 0 prediction decomposition). *E*[*f*(*x*)] on the horizontal axis is the baseline value of the model. The red bars represent features with positive SHAP values, which push the predicted value up. The blue bars represent features with negative SHAP values, which pull the predicted value down. The length of each bar represents the absolute magnitude of the contribution of that feature. After all the bars are accumulated, the final value reaches *f*(*x*) at the top of the figure, representing the final model prediction value of this breeding line.

Then, a waterfall plot was used to precisely decompose the prediction of a single sample (breeding line) at a micro level. The waterfall plot is an important tool for interpreting individual predictions. It clearly shows how each SNP gradually pushes the prediction towards the result, starting from the model’s baseline value. Taking breeding line 0 (referring to the 1st breeding line in the original dataset) as an example in Figure 10, it can be seen that the four SNPs with red colors, i.e., S7B_194607907, S1B_555055859, S1A_8118289, and S2A_20855466, have the strongest positive predictive ability for the GEBV of wheat yield trait. The SNPs with blue color, such as S4B_38624956, S4B_425283056, etc., have the strongest negative predictive ability. The remaining features all have negative predictive ability and are generally weak.

**Figure 11:**
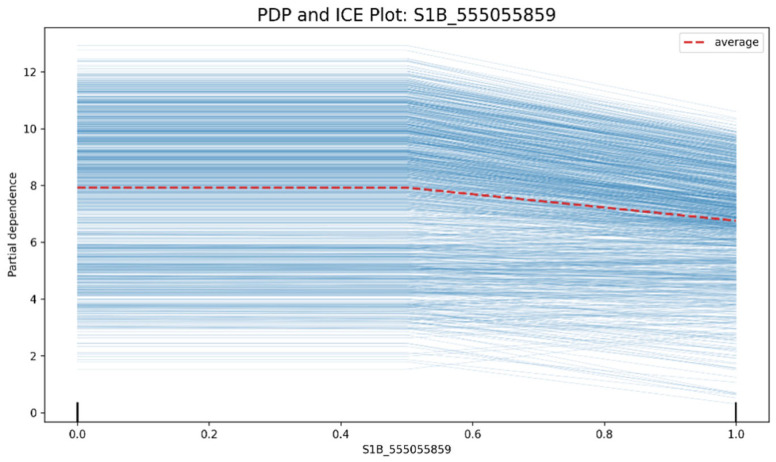
SHAP (PDP) and individual conditional expectation plot (ICE) of SNP S1B_555055859. The thick dashed red line is the PDP, and each thin line represents the ICE curve of a sample. This allows us to see whether there are significant differences in the impact of features on different samples (i.e., whether there are heterogeneous effects). If all the thin lines are roughly parallel, it indicates that the impact of the feature is relatively consistent; if the thin lines cross and are chaotic, it indicates a strong interaction effect.

Finally, combined with the partial dependence plot (PDP) and individual conditional expectation (ICE) plots (Figure 11), the average and individual effects of SNPs were verified. The PDP shows the impact of SNPs on the average GEBV prediction results of the model, and the ICE plot evaluates the impact of SNPs on the prediction results of each sample (breeding line). Taking SNP S1B_555055859 as an example (Figure 11), when the allele value of S1B_555055859 is 0, this feature has a relatively high ability to predict wheat yield (PD value = 8). Currently, all the thin lines are roughly parallel, indicating that the impact of feature S1B_555055859 on different samples is relatively consistent. However, when its allele value is 1, the prediction ability drops to 7. At the same time, the thin lines cross and are chaotic, indicating a strong interaction effect. Further analysis using the dependence plot (Figure 9) is required [32].

## 4. Discussion

### 4.1. GRE Helps Simplify the Data Structure and Improve the Prediction Accuracy of GS

The results of this paper show that the SNP subset obtained by GRE, i.e., the joint feature selection based on GWAS and RF, has a good ability to improve the GEBV prediction of wheat yield traits (Supplementary Tables S2 and S3, Figure 7B). This is also consistent with the conclusions of previous similar studies [13,14,15]. This may be because, theoretically, GWAS and random forest are complementary. GWAS captures the major QTLs through statistical significance, and random forest identifies the interacting markers through non-linear modeling. The intersection subset considers both biological significance and prediction contribution [11,33]. At the same time, we also found that when only considering a single subset, that is, without calculating the intersection and union, the subset obtained by GWAS is superior to the subset obtained by random forest in many scenarios (Supplementary Tables S2 and S3, Figure 7B). The possible reasons are as follows. GWAS adopts the FarmCPU model. The FarmCPU model uses an iterative strategy, combining the advantages of the fixed-effect model and the random-effect model. First, it uses GLM to screen potentially associated loci, and incorporate the screened significant loci as covariates into the MLM loop iteration until the model is stable. This method not only maintains the ability of MLM to control false positives, but also avoids the over-correction problem and significantly improves the statistical power. FarmCPU uses an efficient GLM, and, at the same time, increases the statistical power and reduces false positives through an iterative model selection method. Nevertheless, random forest is still a good complement, mainly because it is an integrated learning algorithm with good robustness. It can adopt the democratic strategy of the minority obeying the majority, it can overcome the influence of individual factors more effectively, and, thus, it can make decision-making more objective and fairer.

### 4.2. Leveraging the Complementary Performance Advantages of the GS Model Can Enhance Prediction Capabilities

In this paper, we simultaneously adopted non-linear models (XGBoost, random forest, LightGBM, SVM) and linear models (GBLUP, ElasticNet) as GS models for comparative research. The results showed that overall, the prediction accuracy of XGBoost, random forest, and LightGBM in non-linear models (average PCC > 0.847) was higher than that of ElasticNet (average PCC 0.84) and GBLUP (average PCC 0.82) in linear models on most subsets (Supplementary Tables S2 and S3, Figure 7B). SVM performed well on small subsets (<2000 SNPs) but was unstable on large subsets (>2000 SNPs) (Figure 7A), possibly because more refined improvements are needed with regard to the model hyperparameter-tuning process. Nevertheless, in genomic selection, linear models (GBLUP, ElasticNet) and non-linear models (XGBoost, random forest, etc.) have their respective advantages and disadvantages, and are suitable for different scenarios [6]. The core advantages of linear models are high efficiency, stability, and strong interpretability, including: (1) They can easily handle millions of markers and large samples in calculations. GBLUP compresses information through the genomic relationship matrix, and its efficiency is higher than that of non-linear models; (2) They can directly estimate marker effects, which is in line with the logic of additive effects in quantitative genetics and is convenient for breeding practice applications; (3) They have high stability and are not prone to over-fitting in small samples or when there is marker redundancy. However, they cannot capture non-linear relationships and gene interactions, have strict assumptions about the distribution of marker effects, and are difficult to deal with traits with complex genetic regulation. The core advantage of non-linear models is their ability to describe complex effects. Tree models capture non-linear and interaction effects through splitting rules, and SVM captures them through kernel functions. They have loose requirements for data distribution, tolerate missing values and outliers, and can also output feature importance to assist in marker screening [22,25]. However, they have high computational costs, and their efficiency is much lower than that of linear models when dealing with high-dimensional markers. They are prone to over-fitting, require large samples for support, and the hyperparameter tuning is complex. Therefore, in wheat, the GS problem with huge genotype data but generally small sample sizes, research can be carried out by integrating models to balance the advantages of both. For example, linear models (such as GBLUP) can be used as benchmark tools for genomic selection to conduct benchmark analysis in scenarios with large samples, mainly additive effects, or when the genetic mechanism needs to be explained. At the same time, the researchers should consider adding dimensionality reduction and non-linear models for comparative research. Especially when the samples are sufficient and the trait inheritance is complex, the accuracy may be improved. Also, balance the computational cost and model interpretability to finally obtain a well-performing and highly efficient GS model suitable for the situation.

### 4.3. An Optimal Value for the Number of Significant SNPs in Wheat Yields Traits

This study found that for the significant SNPs of wheat yield traits in GS, the fewer the number of SNPs, the better it is necessarily. For example, when the number of SNPs is lower than 100, the prediction accuracy is significantly lower than that using the whole set (Figure 4 and Figure 7B, Supplementary Table S2), indicating that the number of significant SNPs for wheat yield trait should not be too small; otherwise, a lot of information will be missed. However, a greater number of SNPs is not necessarily better either. For example, when the number of subsets reaches hundreds or thousands, the PCC is instead higher than that using the whole SNPs (Figure 4 and Figure 7B, Supplementary Table S2). Therefore, there may be a compromise point, such as in the hundreds level (about 383 SNPs found in this study), to the thousands level (about 1000 SNPs). This is similar to the existing research results. Lell et al. showed that when there are approximately 4000 SNPs, the GS prediction accuracy reaches a relatively optimal level [34]. Meyenberg et al. found that even using a low density (about 500) of SNP markers can obtain robust results, providing the possibility of reducing the cost of GS [35]. In this study, a highly sensitive subset with an order of magnitude of hundreds (such as 383) SNPs was extracted from 11,089 SNPs using the joint dimensionality reduction method, which can maintain a high GEBV prediction accuracy, indicating that there is a large amount of redundant information in the wheat genome. It is necessary to continuously explore high-efficiency SNP subsets to improve the GS prediction accuracy. Therefore, adopting an appropriate dimensionality reduction strategy to streamline SNPs is crucial for improving the prediction accuracy of the GS model. It can effectively reduce the large amount of redundant information in the wheat genome, eliminate the false SNPs, retain the true ones, and help breeders discover the significant SNPs that truly affect wheat yield. However, since no GS algorithm has emerged that can perform optimally in the GEBV prediction of all species or traits [6], more verification is still needed for different species or traits, such as key traits like wheat yield and disease resistance.

### 4.4. GRE’s SHAP Techniques Help Identify Significant SNPs in Wheat Yield Traits

In response to the interpretability problem of machine learning algorithms, this study fully explores the SHAP post-hoc interpretation techniques for analyzing the complex quantitative relationship between GS phenotypic and genotypic data. The SHAP technique is based on game theory and the theory of model interpretability [31]. The scientific logic and advantages of each link are as follows:

(1) In the SHAP value calculation, the core basis is the axiomatic system of the Shapley value. By fairly distributing the contribution of each SNP marker to yield prediction, the “black-box” problem of XGBoost is solved. The advantage is that it provides a unified interpretation framework for non-linear models, ensuring that the quantification of the effect of each SNP considers not only its effect but also its interaction contribution with other markers, which is in line with the genetic law of polygenic interaction in the wheat genome.

(2) In the summary graph (Beeswarm + global importance), the Beeswarm graph simultaneously presents the global importance and effect direction (red/blue) of SNPs through the size (vertical axis) and distribution (horizontal axis) of SHAP values. The scientific basis is the cumulative impact of features on prediction bias. The advantage is that it can quickly locate the significant SNPs affecting yield (such as markers with large absolute effect values) and intuitively display their promoting/inhibiting effects on yield, which is suitable for screening candidate markers at the whole-genome level.

(3) In the dependence graph, based on the non-linear relationship between feature values and SHAP values, the interaction effects between SNPs are revealed (such as the effect of one SNP changing with the genotype of another SNP). The scientific basis is the epistatic regulation mechanism of complex traits. The advantage is that it breaks through the limitations of linear models and captures the synergistic/antagonistic effects among genes related to wheat yield. For example, a certain marker significantly affects yield only under a specific genetic background.

(4) In the waterfall plot, by decomposing the predicted value of a single wheat sample (from the baseline to the result), the contribution of each SNP is quantified based on the additivity of the Shapley value. The advantage is to achieve precise interpretation at the micro-level. For example, the reason why the predicted yield of a certain plant is high may be due to the superposition of the positive effects of three to five major SNPs, providing a molecular basis for single-plant selection.

(5) In the combined PDP/ICE graphs, the PDP shows the average marginal effect of SNPs on yield, and the ICE graph presents the individual deviation trend, which is complementary to the SHAP value for verification. The scientific basis is the contrasting relationship between the average effect of the population and individual heterogeneity. The advantage is that it not only confirms the overall influence trend of significant SNPs (such as a certain allele generally increasing yield) but also identifies special samples (such as a certain plant showing a reverse effect due to the genotype combination), enhancing the reliability of the interpretation. This process deeply analyzes the prediction logic of XGBoost for wheat yield from the global to the individual, from population trends to interaction effects. It not only retains the advantage of non-linear models in capturing complex genetic effects but also connects the biological significance of genotypes and phenotypes through interpretability techniques.

### 4.5. Limitations

First, in terms of the representativeness of the wheat samples used in this study, with only 1768 materials, it may be limited to a specific genetic background and needs to be further verified in more diverse wheat populations [36]. Second, in terms of the integration of environmental factors, G×E modeling was not incorporated, and in the future, it can be combined with environmental omics data. Then, in terms of feature selection bias, GWAS has insufficient detection of small-effect QTLs, and RF may overfit noise markers. Therefore, further research is needed to improve the joint screening mechanism and enhance performance [37]. Finally, in terms of data heterogeneity, the complexity of the wheat hexaploidy genome may lead to inconsistent marker effects in different genetic populations, so further exploration is still required.

## 5. Conclusions

This study systematically addresses improving wheat GS prediction accuracy via a novel framework, GRE, i.e., joint feature screening with GWAS and RF for dimensionality reduction, by utilizing six GS algorithms to integrate linear and non-linear model advantages. Furthermore, the interpretability technology of the SHAP series of models is used to accurately analyze the complex quantitative relationship between wheat yield phenotypes and genotypes at the micro-level of the overall macro-view, the main and interaction effects of SNPs, and the average and individual effects of SNPs, further enriching the interpretability difficulties of the “black box” of machine learning and deep learning models in the GS field, and providing strong support for the continuous improvement and enhancement of GS models. The propsed framework, GRE, in this study will contribute to the screening of significant SNPs for important traits in wheat and other typical economic plants and animals. Furthermore, it also provides technical support for the design and development of intelligent breeding chips, especially for improving the yield of plants and animals worldwide and the sustainable development of agriculture.

## Data Availability

The data used in this study are from Lozada et al. (https://journals.plos.org/plosone/article?id=10.1371/journal.pone.0221603, accessed on 22 September 2025).

## Supplementary Materials

The following supporting information can be downloaded at: https://www.mdpi.com/article/10.3390/genes16101125/s1, Method S1. Model evaluation metrics. Table S1. Hyperparameter Tuning of the Machine Learning GS Model; Table S2. Comparison of the prediction PCC effect between random feature selection and GWAS-RF joint selection; Table S3. Comparison of PCC of Six GS Models on 24 SNP subsets and all SNP dataset; Figure S1. The proportion of PCC improvement of the feature selection strategy GRE compared with random feature selection on subsets of different dimensions.
