# Supplementary material for: GRE: A Framework for Significant SNP Identification Associated with Wheat Yield Leveraging GWAS–Random Forest Joint Feature Selection and Explainable Machine Learning Genomic Selection Algorithm
Source: Genes (Basel). 2025 Sep 24;16(10):1125. doi: 10.3390/genes16101125 (PMC12564831; doi:10.3390/genes16101125)
Supplement: Supplementary file 1 [file genes-16-01125-s001.zip › genes-3861011-supplementary.pdf]

# Supplementary materials

## Supplementary Methods

Method S1 Model evaluation metrics

The Pearson correlation coefficient (PCC) (Equation S1) and the coefficient of determination ($R^2$) (Equation S2) are used to evaluate the prediction accuracy of the model. The PCC is a statistic used to reflect the degree of similarity between two variables. In GS, it can be used to calculate the similarity between the real phenotype (wheat yield) and predicted values of wheat yield (GEBVs). The larger the absolute value of PCC, the stronger the positive or negative correlation. Feature selection can be used to remove redundant features with high correlation. The $R^2$ (Equation S2) serves as the core evaluation index of the regression model, measuring the degree of deviation between the model's predicted values and the true values from different dimensions. $R^2$ is used to measure the degree to which the regression model explains the variation of the dependent variable, and its value ranges from 0 to 1.

The Mean Squared Error (MSE) (Equation S3) and Mean Absolute Error (MAE) (Equation S4) are used to evaluate the model error. MSE reflects the average magnitude of the error between the predicted values and the true values. MAE represents the prediction error, reflecting the degree of error of the predicted values relative to the true values, and more intuitively demonstrating the accuracy of the model's predictions.

$$\rho_{X,\ Y} = \frac{\mathrm{cov}(X,Y)}{\sigma_X \sigma_Y} = \frac{E\left((X-\mu_X)(Y-\mu_Y)\right)}{\sigma_X \sigma_Y} \qquad \text{(S1)}$$

where, $\mathrm{cov}(X,Y)$ represent the covariance of $X$ and $Y$, $\sigma_X$ is the standard deviation(SD) of $X$, $\sigma_Y$ is the SD of $Y$.

$$R^2 = 1 - \frac{\sum_{i=1}^{n}(y_i - \hat{y}_i)^2}{\sum_{i=1}^{n}(y_i - \bar{y})^2} \qquad \text{(S2)}$$

$$MSE = \frac{1}{n}\sum_{i=1}^{n}(y_i - \hat{y}_i)^2 \qquad \text{(S3)}$$

$$MAE = \frac{1}{n}\sum_{i=1}^{n}\left|y_i - \hat{y}_i\right| \qquad \text{(S4)}$$

where, $n$ is the sample size, $y_i$ is the real value of the $i$-th sample. $\hat{y}_i$ is the predicted value of the $i$-th sample, $\bar{y}$ is the average of real values.

## Supplementary Tables

**Table S1.** Hyperparameter Tuning of the Machine Learning GS Model.

| Model | Hyperparameters | Candidate values |
|---|---|---|
| ElasticNet | alpha | (0.001, 10) |
| | l1_ratio | (0, 1) |
| SVM | C | (0.01, 100) |
| | gamma | (0.0001, 1) |
| | epsilon | (0.01, 1) |
| RandomForest | n_estimators | (50, 500) |
| | max_depth | (3, 20) |
| | min_samples_split | (2, 10) |
| LightGBM | n_estimators | (50, 500) |
| | learning_rate | (0.01, 0.3) |
| | max_depth | (3, 10) |
| | num_leaves | (20, 100) |
| XGBoost | n_estimators | (50, 500) |
| | learning_rate | (0.01, 0.3) |
| | max_depth | (3, 10) |
| | gamma | (0, 0.5) |

**Table S2.** Comparison of the prediction PCC effect between random feature selection and GWAS-RF joint selection.

| Dataset | # of SNP① | GBLUP | | ElasticNet | | SVM | | Random Forest | | LightGBM | | XGBoost | |
|---|---|---|---|---|---|---|---|---|---|---|---|---|---|
| | | Random SNPs | GRE SNPs | Random SNPs | GRE SNPs | Random SNPs | GRE SNPs | Random SNPs | GRE SNPs | Random SNPs | GRE SNPs | Random SNPs | GRE SNPs |
| D01_100_10_S1_I | 10 | 0.623 ±0.020 | 0.748 ±0.002 | 0.545 ±0.119 | 0.757 ±0.010 | 0.638 ±0.097 | 0.762 ±0.012 | 0.650 ±0.09 | 0.766 ±0.016 | 0.641 ±0.089 | 0.766 ±0.015 | 0.643 ±0.093 | 0.766 ±0.015 |
| D05_200_17_S1_I | 17 | 0.684 ±0.026 | 0.756 ±0.004 | 0.652 ±0.045 | 0.776 ±0.010 | 0.715 ±0.028 | 0.806 ±0.017 | 0.739 ±0.026 | 0.812 ±0.014 | 0.731 ±0.032 | 0.803 ±0.010 | 0.735 ±0.027 | 0.803 ±0.012 |
| D09_500_43_S1_I | 43 | 0.728 ±0.035 | 0.779 ±0.008 | 0.733 ±0.022 | 0.806 ±0.010 | 0.767 ±0.021 | 0.802 ±0.012 | 0.786 ±0.032 | 0.831 ±0.009 | 0.774 ±0.026 | 0.826 ±0.010 | 0.786 ±0.023 | 0.829 ±0.012 |
| D02_100_100_S2_RF | 100 | 0.754 ±0.030 | 0.810 ±0.010 | 0.769 ±0.020 | 0.819 ±0.011 | 0.801 ±0.022 | 0.821 ±0.008 | 0.801 ±0.028 | 0.840 ±0.007 | 0.796 ±0.027 | 0.836 ±0.010 | 0.795 ±0.027 | 0.835 ±0.011 |
| D03_100_100_S3_GWAS | 100 | /② | 0.824 ±0.002 | / | 0.849 ±0.011 | / | 0.859 ±0.006 | / | 0.854 ±0.009 | / | 0.861 ±0.008 | / | 0.860 ±0.004 |
| D13_1000_120_S1_I | 120 | 0.761 ±0.017 | 0.819 ±0.008 | 0.773 ±0.021 | 0.838 ±0.009 | 0.804 ±0.014 | 0.849 ±0.008 | 0.804 ±0.015 | 0.858 ±0.005 | 0.802 ±0.021 | 0.850 ±0.005 | 0.800 ±0.017 | 0.851 ±0.012 |
| D04_100_190_S4_U | 190 | 0.777 ±0.021 | 0.838 ±0.002 | 0.784 ±0.012 | 0.854 ±0.007 | 0.787 ±0.024 | 0.861 ±0.006 | 0.819 ±0.018 | 0.863 ±0.008 | 0.812 ±0.011 | 0.859 ±0.007 | 0.811 ±0.015 | 0.861 ±0.005 |
| D06_200_200_S2_RF | 200 | 0.776 ±0.013 | 0.817 ±0.002 | 0.790 ±0.013 | 0.829 ±0.008 | 0.818 ±0.012 | 0.832 ±0.008 | 0.818 ±0.011 | 0.858 ±0.008 | 0.818 ±0.013 | 0.849 ±0.007 | 0.815 ±0.010 | 0.846 ±0.008 |
| D07_200_200_S3_GWAS | 200 | / | 0.840 | / | 0.868 | / | 0.870 | / | 0.859 | / | 0.865 | / | 0.863 |

| Dataset | # of SNP① | GBLUP | | ElasticNet | | SVM | | Random Forest | | LightGBM | | XGBoost | |
|---|---|---|---|---|---|---|---|---|---|---|---|---|---|
| | | Random SNPs | GRE SNPs | Random SNPs | GRE SNPs | Random SNPs | GRE SNPs | Random SNPs | GRE SNPs | Random SNPs | GRE SNPs | Random SNPs | GRE SNPs |
| | | | ±0.003 | | ±0.002 | | ±0.004 | | ±0.009 | | ±0.006 | | ±0.003 |
| D08_200_383_S4_U | 383 | 0.794 ±0.011 | 0.845 ±0.005 | 0.802 ±0.009 | 0.865 ±0.002 | 0.805 ±0.007 | 0.873 ±0.006 | 0.835 ±0.007 | 0.863 ±0.006 | 0.825 ±0.005 | 0.863 ±0.003 | 0.827 ±0.008 | 0.864 ±0.005 |
| D17_2000_383_S1_I | 383 | / | 0.834 ±0.008 | / | 0.852 ±0.009 | / | 0.857 ±0.010 | / | 0.855 ±0.006 | / | 0.853 ±0.004 | / | 0.853 ±0.007 |
| D10_500_500_S2_RF | 500 | 0.798 ±0.015 | 0.825 ±0.005 | 0.809 ±0.014 | 0.841 ±0.009 | 0.810 ±0.013 | 0.840 ±0.009 | 0.839 ±0.011 | 0.862 ±0.006 | 0.827 ±0.016 | 0.854 ±0.006 | 0.827 ±0.010 | 0.850 ±0.007 |
| D11_500_500_S3_GWAS | 500 | / | 0.851 ±0.004 | / | 0.859 ±0.006 | / | 0.866 ±0.002 | / | 0.854 ±0.007 | / | 0.863 ±0.006 | / | 0.859 ±0.007 |
| D21_3000_821_S1_I | 821 | 0.801 ±0.020 | 0.845 ±0.006 | 0.811 ±0.010 | 0.858 ±0.013 | 0.809 ±0.011 | 0.861 ±0.007 | 0.845 ±0.014 | 0.859 ±0.008 | 0.838 ±0.016 | 0.855 ±0.010 | 0.833 ±0.013 | 0.856 ±0.006 |
| D12_500_957_S4_U | 957 | 0.808 ±0.005 | 0.853 ±0.008 | 0.813 ±0.012 | 0.861 ±0.005 | 0.805 ±0.007 | 0.857 ±0.011 | 0.843 ±0.006 | 0.862 ±0.007 | 0.839 ±0.008 | 0.860 ±0.007 | 0.834 ±0.007 | 0.860 ±0.007 |
| D14_1000_1000_S2_RF | 1000 | 0.799 ±0.013 | 0.826 ±0.007 | 0.818 ±0.007 | 0.838 ±0.008 | 0.805 ±0.006 | 0.830 ±0.011 | 0.845 ±0.008 | 0.861 ±0.007 | 0.843 ±0.010 | 0.858 ±0.008 | 0.838 ±0.009 | 0.845 ±0.007 |
| D15_1000_1000_S3_GWAS | 1000 | / | 0.856 ±0.001 | / | 0.856 ±0.011 | / | 0.869 ±0.004 | / | 0.858 ±0.009 | / | 0.865 ±0.007 | / | 0.864 ±0.005 |
| D16_1000_1880_S4_U | 1880 | 0.815 ±0.006 | 0.852 ±0.010 | 0.825 ±0.010 | 0.848 ±0.020 | 0.706 ±0.216 | 0.707 ±0.314 | 0.846 ±0.015 | 0.860 ±0.006 | 0.842 ±0.009 | 0.860 ±0.008 | 0.838 ±0.015 | 0.858 ±0.007 |

| Dataset | # of SNP① | GBLUP | | ElasticNet | | SVM | | Random Forest | | LightGBM | | XGBoost | |
|---|---|---|---|---|---|---|---|---|---|---|---|---|---|
| | | Random SNPs | GRE SNPs | Random SNPs | GRE SNPs | Random SNPs | GRE SNPs | Random SNPs | GRE SNPs | Random SNPs | GRE SNPs | Random SNPs | GRE SNPs |
| D18_2000_2000_S2_RF | 2000 | 0.820 ±0.011 | 0.826 ±0.001 | 0.821 ±0.006 | 0.838 ±0.009 | 0.807 ±0.005 | 0.861 ±0.017 | 0.850 ±0.01 | 0.858 ±0.007 | 0.843 ±0.010 | 0.850 ±0.005 | 0.842 ±0.012 | 0.846 ±0.004 |
| D19_2000_2000_S3_GWAS | 2000 | / | 0.855 ±0.006 | / | 0.854 ±0.018 | / | 0.624 ±0.298 | / | 0.855 ±0.008 | / | 0.862 ±0.006 | / | 0.858 ±0.008 |
| D22_3000_3000_S2_RF | 3000 | 0.818 ±0.004 | 0.830 ±0.004 | 0.830 ±0.006 | 0.839 ±0.006 | 0.829 ±0.009 | 0.838 ±0.004 | 0.848 ±0.012 | 0.856 ±0.008 | 0.845 ±0.013 | 0.850 ±0.003 | 0.841 ±0.009 | 0.848 ±0.005 |
| D23_3000_3000_S3_GWAS | 3000 | / | 0.861 ±0.006 | / | 0.853 ±0.010 | / | 0.574 ±0.362 | / | 0.857 ±0.008 | / | 0.861 ±0.007 | / | 0.860 ±0.009 |
| D20_2000_3617_S4_U | 3617 | 0.830 ±0.004 | 0.852 ±0.004 | 0.835 ±0.007 | 0.841 ±0.009 | 0.673 ±0.327 | 0.862 ±0.004 | 0.849 ±0.01 | 0.856 ±0.007 | 0.842 ±0.008 | 0.857 ±0.005 | 0.838 ±0.007 | 0.852 ±0.007 |
| D24_3000_5179_S4_U | 5179 | 0.827 ±0.002 | 0.852 ±0.002 | 0.835 ±0.004 | 0.856 ±0.006 | 0.838 ±0.002 | 0.862 ±0.003 | 0.852 ±0.009 | 0.856 ±0.006 | 0.843 ±0.008 | 0.852 ±0.006 | 0.840 ±0.009 | 0.849 ±0.007 |
| D25_D2_wheat (all SNPs) | 11089 | / | 0.832 ±0.003 | / | 0.837 ±0.008 | / | 0.848 ±0.006 | / | 0.853 ±0.007 | / | 0.851 ±0.006 | / | 0.845 ±0.008 |

Note: The number of SNPs in the two subsets is 383, one of them is the union of the TOP200 of RF and GWAS, and the second is the intersection of the TOP2000 of RF and GWAS, so the 383-subset is randomly selected only once. Similarly, for the TOP100 of RF and the TOP100 of GWAS, this paper randomly selects 100 SNP features only once and compares them together, and so on. Mean ± SD is the PCC's mean value and standard deviation. ① "# of SNP" represents the number of SNPs. ② The symbol "/" indicates that when randomly selecting features, if there is a repetition at this order of magnitude, only the subset of this order of magnitude is selected once.

**Table S3.** Comparison of PCC of Six GS Models on 24 SNP subsets and all SNP dataset.

| Dataset | # SNPs | GBLUP | Elastic Net | SVM | Random Forest | LightGBM | XGBoost | Mean of subsets* |
|---|---|---|---|---|---|---|---|---|
| D01_100_10_S1_I | 10 | 0.748 | 0.757 | 0.762 | 0.766 | 0.766 | 0.766 | 0.761 |
| D05_200_17_S1_I | 17 | 0.756 | 0.776 | 0.806 | 0.812 | 0.803 | 0.803 | 0.790 |
| D09_500_43_S1_I | 43 | 0.779 | 0.806 | 0.802 | 0.831 | 0.826 | 0.829 | 0.814 |
| D02_100_100_S2_RF | 100 | 0.810 | 0.819 | 0.821 | 0.840 | 0.836 | 0.835 | 0.828 |
| D03_100_100_S3_GWAS | 100 | 0.824 | 0.849 | 0.859 | 0.854 | 0.861 | 0.860 | 0.850 |
| D13_1000_120_S1_I | 120 | 0.819 | 0.838 | 0.849 | 0.858 | 0.850 | 0.851 | 0.843 |
| D04_100_190_S4_U | 190 | 0.838 | 0.854 | 0.861 | 0.863 | 0.859 | 0.861 | 0.855 |
| D06_200_200_S2_RF | 200 | 0.817 | 0.829 | 0.832 | 0.858 | 0.849 | 0.846 | 0.840 |
| D07_200_200_S3_GWAS | 200 | 0.840 | 0.868 | 0.870 | 0.859 | 0.865 | 0.863 | 0.859 |
| D08_200_383_S4_U | 383 | 0.845 | 0.865 | 0.873 | 0.863 | 0.863 | 0.864 | 0.860 |
| D17_2000_383_S1_I | 383 | 0.834 | 0.852 | 0.857 | 0.855 | 0.853 | 0.853 | 0.849 |
| D10_500_500_S2_RF | 500 | 0.825 | 0.841 | 0.840 | 0.862 | 0.854 | 0.850 | 0.846 |
| D11_500_500_S3_GWAS | 500 | 0.851 | 0.859 | 0.866 | 0.854 | 0.863 | 0.859 | 0.857 |
| D21_3000_821_S1_I | 821 | 0.845 | 0.858 | 0.861 | 0.859 | 0.855 | 0.856 | 0.855 |

Dataset # SNPs GBLUP Elastic Net SVM Random Forest LightGBM XGBoost Mean of subsets*

| | | | | | | | | |
|---|---|---|---|---|---|---|---|---|
| D12_500_957_S4_U | 957 | 0.853 | 0.861 | 0.857 | 0.862 | 0.860 | 0.860 | 0.859 |
| D14_1000_1000_S2_RF | 1000 | 0.826 | 0.838 | 0.830 | 0.861 | 0.858 | 0.845 | 0.846 |
| D15_1000_1000_S3_GWAS | 1000 | 0.856 | 0.856 | 0.869 | 0.858 | 0.865 | 0.864 | 0.860 |
| D16_1000_1880_S4_U | 1880 | 0.852 | 0.848 | 0.707 | 0.860 | 0.860 | 0.858 | 0.856 |
| D18_2000_2000_S2_RF | 2000 | 0.826 | 0.838 | 0.861 | 0.858 | 0.850 | 0.846 | 0.844 |
| D19_2000_2000_S3_GWAS | 2000 | 0.855 | 0.854 | 0.624 | 0.855 | 0.862 | 0.858 | 0.857 |
| D22_3000_3000_S2_RF | 3000 | 0.830 | 0.839 | 0.838 | 0.856 | 0.850 | 0.848 | 0.845 |
| D23_3000_3000_S3_GWAS | 3000 | 0.861 | 0.853 | 0.574 | 0.857 | 0.861 | 0.860 | 0.858 |
| D20_2000_3617_S4_U | 3617 | 0.852 | 0.841 | 0.862 | 0.856 | 0.857 | 0.852 | 0.852 |
| D24_3000_5179_S4_U | 5179 | 0.852 | 0.856 | 0.862 | 0.856 | 0.852 | 0.849 | 0.853 |
| D25_D2_wheat (all SNPs) | 11089 | 0.832 | 0.837 | 0.848 | 0.853 | 0.851 | 0.845 | 0.844 |
| **Mean of GS models** | | 0.829 | 0.840 | 0.820 | 0.851 | 0.849 | 0.847 | 0.843 |

# SNPs, the number of SNPs. * Exclude the SVM model because of its unstably.

## Supplementary Figures

### Figure S1

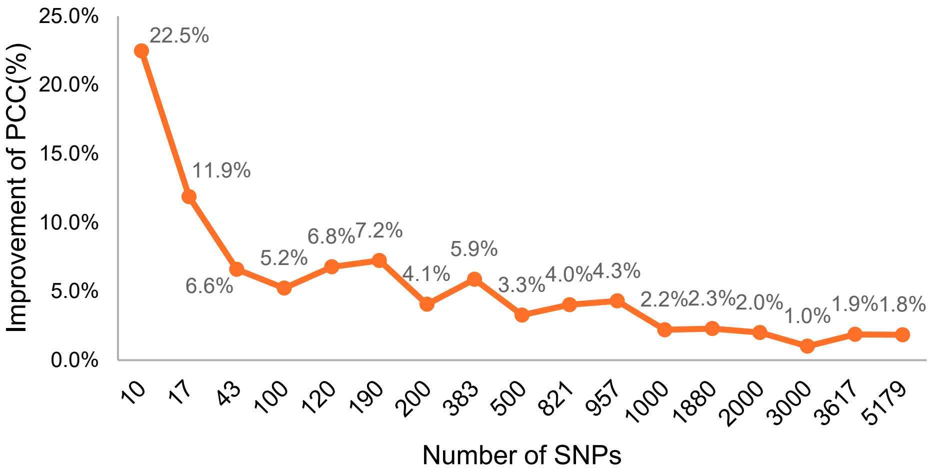


Figure S1. The proportion of PCC improvement of the feature selection strategy GRE compared with random feature selection on subsets of different dimensions
